# Levels of Physical Activity, Obesity and Related Factors in Young Adults Aged 18–30 During 2009–2017

**DOI:** 10.3390/ijerph16204033

**Published:** 2019-10-21

**Authors:** José Alberto Laredo-Aguilera, Ana Isabel Cobo-Cuenca, Esmeralda Santacruz-Salas, María Manuela Martins, María Aurora Rodríguez-Borrego, Pablo Jesús López-Soto, Juan Manuel Carmona-Torres

**Affiliations:** 1 Department of Nursing, Physiotherapy and Occupational Therapy, Universidad de Castilla-La Mancha, 45600 Talavera de la Reina, 45071 Toledo, Spain; jalaredo@hotmail.com (J.A.L.-A.); esmeralda.santacruz@uclm.es (E.S.-S.); juanmanuel.carmona@uclm.es (J.M.C.-T.); 2 Grupo de Investigación Multidisciplinar en Cuidados (IMCU), 45071 Toledo, Spain; 3 Instituto Maimónides de Investigación Biomédica de Córdoba (IMIBIC), 14004 Córdoba, Spain; en1robom@uco.es (M.A.R.-B.); n82losop@uco.es (P.J.L.-S.); 4 Escola Superior de Enfermagem/CINTESIS, 4200-072 Porto, Portugal; mmartins@esenf.pt; 5 Universidad de Córdoba, 14004 Córdoba, Spain; 6 Hospital Universitario Reina Sofía de Córdoba, 14004 Córdoba, Spain

**Keywords:** young adults, physical activity, obesity, overweight, BMI

## Abstract

The objective of this study was to analyze the temporal trend of physical activity and body mass index in young adults aged 18–30 in Spain and to ascertain their relationship with sociodemographic and psychosocial variables in the period of 2009–2017. Methods: A descriptive study with a sample of 10,061 young adults aged 18–30 years was performed. The data were obtained from the European Health Survey in Spain in 2009 and 2014 and the National Health Survey in 2011/2012 and 2017. The chi-square test was used for qualitative variables, and multiple linear regression analysis was performed for physical activity. Results: Sedentary levels had decreased in 2017 as compared to 2011/2012 (*p* < 0.001); smokers were more sedentary than non-smokers (*p* < 0.001); men were more active than women (*p* < 0.001); and the year with the highest physical activity was 2014. Body mass index in the total sample increased from 2009 to 2017 (*p* < 0.01), showing a significant increase in obesity in women (*p* < 0.05) and no difference in men (*p* ≥ 0.05). Conclusions: In the period 2011/2012–2017, the sedentary lifestyle of young adults was reduced and physical activity was increased, with men being more active than women.

## 1. Introduction

There is increasing evidence that physical activity (PA) has very beneficial effects for people of all ages, and the sooner these habits are acquired, the greater the benefits, contributing to maintenance of a person’s health as they advance in age [1]. 

Among different health disorders, there is obesity. Currently, 70% of adults with obesity have acquired it in adulthood [2]. On the other hand, anxiety and depression have been directly related to weight gain in young adults [3]. In Spain, the population aged 18–30 years amounts to 5.3 million [4], which gives an idea of the magnitude of the problem. Body mass index (BMI) and obesity are related to levels of PA [5].

Mechanization in all areas has affected the way of life, both in the workplace (reducing energy expenditure) and at home (increasing sedentary lifestyle) [6]. Sedentary behaviors, such as passive transport, have also been associated with increased obesity [7]. Physical activity is also important as an appetite regulator, observing that people with low levels of PA have higher levels of food cravings than people with high levels of PA [8]. It has been proven that both levels of PA and sociodemographic and psychosocial characteristics influence obesity [9]. In a 32-year longitudinal study, it was observed that twins with different lifestyles and sociodemographic and psychosocial differences had significant differences in body weight, BMI and fat percentage, with the lowest results in those that had the highest levels of PA [9].

A reduction in mobility is associated with lower levels of PA and higher mortality from cardiovascular disease [10]. The prevention of coronary heart disease requires a better understanding of risk factors and healthy lifestyle behaviors from an early age [11]. Several epidemiological studies [11,12,13] reflect that there are cardiovascular benefits and a decrease in the risk of cardiovascular diseases and deaths when practicing the recommended levels of PA [11]. These same authors [12,13] demonstrated that greater intensity and higher activity sessions have an association with reductions in risk and mortality from vascular diseases in white men.

According to Haskell et al. [14], PA in healthy people should be 150 min at moderate intensity or 60 mins at high intensity per week for health maintenance. Furthermore, the activity has to be for periods of 10 min at a minimum [14]. 

Andersson et al. [15] noted that maintaining recommended levels of PA showed a reduction of 1.8 years in vascular ageing. On the other hand, it is estimated that if adults reduced their sitting by 3 h or more per day, it would increase their life expectancy by two years [16]. Shah et al. [17] noted that every minute that PA was reduced in young adults over a period of seven years was significantly associated with cardiovascular disease and long-term mortality risk, but, if physical capacity was increased, heart failure was reduced. Other authors have emphasized that maintaining PA habits improves health status over the years and favors active ageing [18].

Previously, obesity has been mentioned as a health problem that can arise as a result of a decrease in PA [5]. Obesity is, in turn, an added health complication in people with chronic diseases and inflammatory processes [19]. 

The prevalence of overweight/obesity and its concomitant health risks are almost universal [20]. The World Health Organization aims to stop its increase by 2025 [21]. For this, it is necessary to quantify levels of overweight and obesity and to develop action policies in those places where it is most required [22]. 

Obesity has not decreased significantly in any country worldwide in the last 33 years [20]. However, it is expected that developed countries have reached the maximum point, while obesity in developing countries will not increase by more than 40%; the truth, however, is that obesity is continuing to increase [20]. 

Another health disorder directly or indirectly related to decreased exercise is problems with sleep [23,24]. Problems including alterations in sleep quality or quantity affect up to 40% of young adults [23] and have an inverse association with sedentary lifestyle and obesity in young adults [24]. Increasing hours of sleep on weekends compared to sleep time during the week is associated with an increase in BMI and adiposity [5]. 

Physical inactivity and poor dietary intake or poor dietary habits are related to alterations in wellbeing, weight and health in general [25], all of which are associated with risks of ischemic heart disease, stroke, type 2 diabetes, depression and some types of cancer [25]. 

Another study [18] confirms the effects of PA levels, sedentary lifestyle, overweight and obesity, and the importance of maintaining PA habits in adulthood and in older adults.

The increase in rates of overweight and obesity in the young population [16], together with the health problems they cause during adulthood [15], creates the need to analyze the sociodemographic and psychosocial factors that are associated with these problems in a young population. It has been observed how sociodemographic and psychosocial variables also influence obesity [6,7,9]. On the other hand, there is controversy as to whether physical activity and energy expenditure can provide a solution to obesity; there are authors who are against their contribution [26,27,28] and in their favor [8,29,30,31]. In the present study, the authors considered as an object of interest the analysis of the Spanish population aged 18–30, as it is the first stage of adulthood and where it is believed that there is a great possibility of changing life habits [32,33]. Based on the aforementioned, the aim of the study was to know the influence and relationship of PA on BMI and sociodemographic and psychosocial characteristics in young Spanish adults aged 18–30 in the period of 2009–2017.

## 2. Materials and Methods

### 2.1. Participants and Design

A cross-sectional study was conducted in different years (2009, 2011/2012, 2014 and 2017) with different samples and registration of young adults aged 18–30 residing in Spain using records from the European Health Survey in Spain (EHSS) in 2009 [34] and 2014 [35] and the National Health Survey (NHS) in 2011/2012 [36] and 2017 [37]. The surveys were conducted by the National Statistics Institute (INE, abbreviation in Spanish) and the Ministry of Health, Social Services and Equality (MSSI, abbreviation in Spanish). In both surveys, personal interviews were conducted by the INE and MSSI using multi-stage probabilistic sampling by municipalities (first stage), sections (second stage) and individuals (third stage). Participants were selected by random sampling and fees based on sex and age. The inclusion criteria, for the data analyzed in the present study, were age 18–30 and a resident of Spain during the years of the surveys. The exclusion criteria were age under 18 or over 30 and inability to respond to the interview, whether due to disability, illness, ignorance of the language or any other circumstance. The data obtained from the surveys were extracted from web pages of the INE and MSSI in the form of anonymized microdata, thus no authorization was required for their use. According to Spanish law, no report from the ethics committee is required to use anonymous and public data from these institutions.

For the present study, all the records of young adults aged 18–30 were selected. The total sample of participants numbered 10,061: 2879 were from 2009, 2637 from 2011/2012, 2350 from 2014 and 2195 from 2017.

### 2.2. Outcome Measures

The dependent variable was the frequency of PA, which could be sedentary, perform physical activity occasionally, perform physical activity several times a month and perform physical activity several times a week.

The independent variables were: the year of the survey (quantitative variable), 2009, 2011/2012, 2014 or 2017; age (quantitative variable); sex (categorical), male or female; nationality (categorical), Spanish or foreigner; smoker (dichotomous), yes or no; marital status (categorical), single, married, divorced, widowed, separated and unifying separated, divorced or widowed in a single variable; educational level (categorical), without studies, primary, secondary, high school/vocational education and training or university; social class (established according to the categories proposed by the Spanish Society of Epidemiology [38]), which was stratified into the three levels of high class (Level I, directors and managers of companies with 10 or more employees and professionals with university degrees; and Level II, directors of companies with less than 10 employees and professionals with college diplomas), medium class (Level III, intermediate occupations, and Level IV, workers in qualified technical occupations), and low class (Level V, primary sector workers; and Level VI, unskilled workers); health self-perception (categorical), psychosocial problems measured by the Goldberg Health Questionnaire (GHQ12); depression (dichotomous) through direct questioning; and the BMI (quantitative variable), which was calculated from self-reported body weight and height.

### 2.3. Statistical Analysis

The quantitative variables are expressed by means of the median and the mode, since they did not follow a normal distribution when performing the Kolmogorov–Smirnov normality test. Qualitative variables (categorical or dichotomous) are expressed by count (*n*) and percentage (%). The categorized variables were compared using chi-square tests for contingency tables; in the case of 2 × 2 tables, the chi-square statistic with Yates correction was used, and, when some expected frequency was ≤5, Fisher’s exact test was applied. Multiple logistic regression was performed to determine the influence of the variables on the frequency with which young adults performed PA. For this regression, it was established that they did not perform PA frequently if they described being sedentary or occasionally performing PA, while performing PA several times a month or several times a week established that they performed PA frequently. The Wald statistic was used, in which variables with *p* ≥ 0.15 were eliminated one by one from the model. Odds ratios (ORs) were calculated with 95% confidence intervals. Statistical significance was considered with a value of *p* < 0.05, and all hypothesis contrasts were bilateral. The statistical program IBM SPSS, version 24 (IBM Corp, Armonk, NY, USA), licensed for the University of Castilla-La Mancha (UCLM) was used for the statistical treatment of the data.

## 3. Results

The total sample of participants in the study numbered 10,061, with an age range of 18–30. The median age was 25, and the mode age was 30. In height, both the median and the mode were 170 cm, and, in terms of weight, a median of 68 kg and a mode of 70 kg were obtained. Of the total sample, 48.6% were men versus 51.4% women, and it was observed that 81.6% were single, 17.3% married and 1.1% widowed, separated or divorced. Young adults who reported a state of health perceived as regular, bad or very bad accounted for 11.2% of the total sample. Of those surveyed, 8.7% reported a slight limitation and 1.3% a severe limitation in the last six months. In turn, 56.5% of young adults reported that worry made them lose a lot of sleep.

Of the sample, 35.8% were smokers, of whom 53.6% were men, and a significant difference was found between sexes (*p* < 0.001). Being constantly overwhelmed and stressed was reported by 60.5% of the sample, of whom women represented 54.3%, with a significant difference between sexes (*p* < 0.001). A feeling that they could not overcome their difficulties was reported by 48.9% of the total sample, which was composed of 47.4% men and 52.6% women (*p* ≥ 0.05). Of the total sample, 39.5% reported feeling unhappy, which was composed of 46.1% men and 53.9% women (*p* < 0.01). A loss of self-confidence was felt by 27.4% of the total sample, which was composed of 46.2% men and 53.8% women (*p* < 0.05). Table 1 shows the sociodemographic characteristics of the sample according to the years of the surveys.

Table 2 shows the anthropometric variables and their relationship with frequency of PA among the young adults studied. It can be seen that men had more PA than women (*p* < 0.001), that the percentage of smokers was lower among those who trained several times a week, and that those who have sedentary behavior are those who smoked the most. Regarding the percentage of people who did PA several times a week, it was observed that, in 2014, there was a higher percentage, with 2011/2012 being the year in which the fewest people did PA several times a week. Therefore, one could say that was the year in which a sedentary lifestyle was most common.

**Figure 1:**
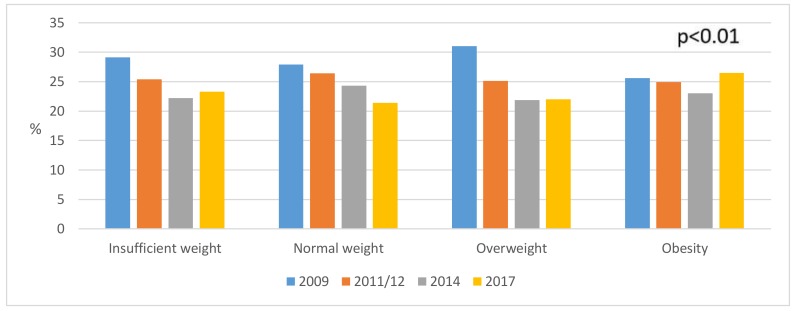
Weight status in the total simple (*n* = 10,061) (2009–2017).

**Figure 2:**
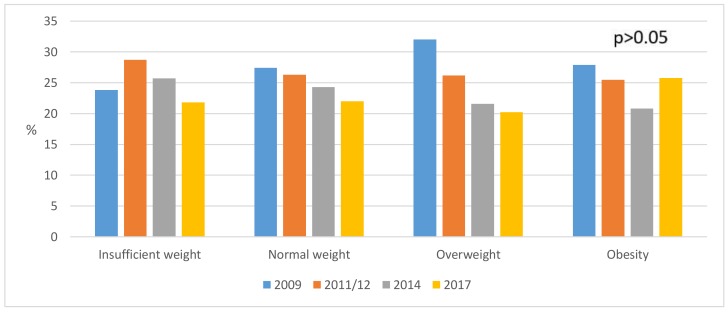
Weight status in young male adults (*n* = 4886) (2009–2017).

**Figure 3:**
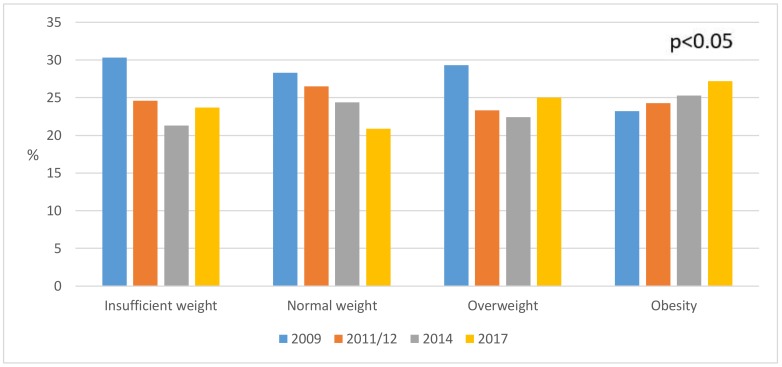
Weight status in young female adults (*n* = 5175) (2009–2017).

The BMI data in the different years of the surveys can be seen in Figure 1, Figure 2 and Figure 3. The BMI of the total sample can be observed in Figure 1 (*p* < 0.01).

**Figure 4:**
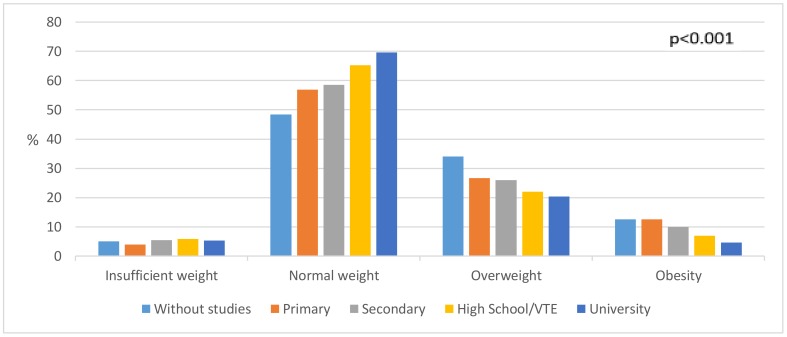
Distribution of weight status at the educational level (2009–2017). VTE, Vocational Education and Training.

The BMI of men is presented in Figure 2 (*p* > 0.05), which shows how the percentage of overweight men decreased from a value of 32% in 2009 to 20.2% in 2017. The BMI of women is shown in Figure 3 (*p* < 0.05), which shows the percentage of obese women rose from 23.2% in 2009 to 27.2% in 2017. BMI and its relation to educational level can be seen in Figure 4 (*p* < 0.001), where 69.7% of people with university studies had a normal weight as compared to 48.4% of people without studies.

Of the total sample, 5.4% were underweight, 63.7% were of normal weight and 30.9% overweight or obese. Looking at the distribution by weight, 8% of the participants in the surveys weighed less than 50 kg, 51.8% weighed 51–70 kg, 33.1% weighed 71–90 kg, 5.9% weighed 91–110 kg and 1.2% weighed more than 110 kg. Table 3 shows the relationship between BMI and different anthropometric variables in the sample.

The logistic regression analysis presented in Table 4 shows the frequency of PA and its association with normal weight (OR 1.66, 95% CI 1.25–2.20, *p* = 0.001), alcohol consumption (1–2 times/week (OR 1.97, 95% CI 1.47–2.63, *p* < 0.001) and 1–3 times/month (OR 1.56, 95 CI 1.22–2, *p* < 0.001)), being male (OR 2.63, 95% CI 2.09–3.32, *p* < 0.001), being single (OR 2.11, 95% CI 1.58–2.8, *p* < 0.001), university studies (OR 2.66, 95% CI 0.99–7.18, *p* = 0.054), feeling more happy than usual (OR 1.97, 95% CI 1.09–3.58, *p* = 0.026), feeling happy as usual (OR 1.55, 95% CI 0.91–2.66, *p* = 0.110), Social Classes I and II (OR 1.34, 95% CI 1.02–1.78, *p* = 0.042), Social Classes II and IV (OR 1.35, 95% CI 1.08–1.70, *p* = 0.008), smoking (OR 1.66, 95% CI 1.33–2.08, *p* < 0.001) and weighing 71–90 kg (OR 1.98, 95% CI 0.83–4.70, *p* = 0.122).

## 4. Discussion

The aim of the study was to know the influence and relationship of PA on BMI and sociodemographic and psychosocial characteristics in young Spanish adults aged 18–30 during 2009–2017. The study found that obesity increased according to the age of the respondents, rising from 21.9% between 18 and 22 years old to 50.3% between 27 and 30 years old. Another finding was that a sedentary lifestyle decreased considerably from 42.1% in 2011/2012 to 29.5% in 2017.

A high BMI and depression are two current problems in society carrying a significant burden in terms of disease, functional disability and mortality [3,19,20,22,39,40]. In line with our findings, it has been found that obesity increases the risk of depression [41,42] and that the reverse is also true [43], with there being a bidirectional relationship. Physical activity seems to have a significant relationship with depression [39,44,45] and with overweight and obesity [5,46]. A possible solution to improve both pathologies is PA. This study shows that obese people have more than twice as much depression as those with normal weight. In sedentary youth, there is the highest rate of obesity and depression. Trends have been observed that depressed obese people do less PA than obese people without depression [39].

In the different years of the study, sedentary lifestyle significantly reduced and frequency of PA increased significantly to several times a week among young adults between 2011/2012 and 2017. However, there was also an increase in obesity between 2011/2012 and 2017; these results are similar to other studies [47,48]. This could be due to the fact that obesity in the total sample is influenced by obesity among women, which has increased progressively in every year of the survey. In men, obesity decreased in 2014 (the year in which obesity fell in the total sample), when PA increased to several times per week, and they had a less sedentary lifestyle; 2011/2012 and 2017 showed similar values. The incidence of obesity was stable in men and increased in women, which may be due to men doing 2.63 times more PA than women and is similar to data in another recent study [48].

Among the results presented, it is stated that PA in the young adults surveyed is related to levels of happiness, although other studies refer to the relationship between PA and quality of life, self-esteem and affection [9,49]. With regard to happiness, failure to maintain adequate PA is associated with lower levels of mental health, including depression [50]. In our study, sedentary lifestyle and obesity are associated with a higher percentage of respondents who reported having suffered depression, a finding that is also corroborated in the literature [39,44,45,51].

The differences in relation to social class are also reflected in the figures of obesity and overweight in Social Classes I and II and in Social Classes V and VI, with the values of Social Classes V and VI (5.7 and 3.9, respectively) greater than Social Classes I and II. In this case, it should be said that Social Classes V and VI showed the highest percentage of respondents. There are studies that show results similar to those found in this work, reflecting the relationship of obesity with social class and parents’ BMI status [52,53,54]. In addition, it can be seen how obesity is related to PA, since Social Classes I and II and Social Classes III and IV have ORs of 1.34 and 1.35, respectively, in relation to doing more PA than Social Classes V and VI. A similar situation occurs with the educational level of the respondents, in which it is seen that those who have university studies do 2.66 times more PA than respondents without studies. Similarly, a higher educational level correlates with lower obesity, lower overweight, lower underweight and higher normal weight, similar results to those obtained by Peterson [55].

Alcohol consumption among young adults is related to BMI. It is observed that young adults with obesity have a higher rate of alcohol consumption as compared to those with normal weight, which is similar to the study by Monteiro et al. [56]. In relation to alcohol consumption and the frequency of PA of young adults, it is observed that those who do PA consume more alcohol than those who do not, coinciding with the findings of a recent review [57].

The finding of a relationship between a healthy lifestyle including PA and an unhealthy lifestyle including alcohol consumption may be surprising, and it may be due to a more active social life, as argued by other authors [54,58]. However, we observe in this work how, with tobacco, we obtain opposite values, since sedentary young adults are 1.66 times more likely to smoke than those who do PA.

Physical activity programs, including programs involving parents in healthy habits for their children [59], the use of mobile PA applications [60], recreational activities that require movement [61] and virtual reality implementation [62], among others, are interventions that offer positive results in increasing levels of PA and reducing obesity in the young population.

## 5. Limitations

A limitation of this study is that PA was not objectively quantified with accelerometers or similar devices, as it was collected through self-reported questionnaires. With more objective instruments, the data collected would have greater validity and could avoid a possible altered perception by respondents. Another limitation is that dietary control data were not available for association with BMI, which was calculated by weight and self-referenced height; therefore, it was not measured with sensitivity devices indicating body composition, such as the fat mass and muscle mass of participants, which would have provided more detailed information on participants’ body composition and the association with PA. With a more precise control of the diet and evaluation of body composition, it could be known if the increase in weight produced in 2017 was due to fat mass or muscle mass. Therefore, it would be interesting to carry out future studies that took these characteristics into account.

However, among the strengths of the study is the sample size, which is nationally representative and updated evidence on the situation of young adults in Spain, in relation to obesity and PA, as well as the circumstances that concur, among which that PA does help reduce obesity. All of this will allow for coherent intervention patterns.

## 6. Conclusions

In the period between 2011/2012 and 2017, sedentary lifestyle in young adults was reduced and PA was increased, with men being more active than women.

In this study, the realization of PA several times a week was associated with being a man, being a non-smoker, having a very good perception of one’s health, having good mental health and being happy and self-confident.

Obesity was associated with young adults between 27 and 30 years old, who belong to Social Class V or VI, lead a sedentary lifestyle and are without studies. Over the study period, obesity was increased in women and decreased in men.

As implications for practice, the data indicate the need to propose programs that promote PA and diet control in young adults, aiming at a higher uptake among young women to curb the increase in overweight and obesity levels in this population in order to try to reverse them in later adulthood and achieve habits to maintain a healthy state throughout life.

## Figures and Tables

**Table 1:** Sociodemographic characteristics of Spanish people 18–30 (n = 10,061) in the period 2009–2017.

Characteristics	2009 n = 2879 (%)	2011/2012 n = 2637 (%)	2014 n = 2350 (%)	2017 n = 2195 (%)	*p*
*Age*	24.81 (SD 3.8)	24.62(SD 3.76)	24.68 (SD 3.76)	24.37 (SD 3.82)	0.15
*Sex*					
Men	1414 (49.1)	1290 (48.9)	1126 (47.9)	1056 (48.6)	0.787
Women	1465 (50.9)	1347 (51.1)	1224 (52.1)	1139 (51.4)	
*Smoke*					
Yes	1127 (40.3)	978 (37.1)	803 (34.2)	667 (30.4)	<0.001
No	1671 (59.7)	1657 (62.9)	1545 (65.8)	1527 (69.6)	
*Nationality*					
Spanish	2478 (86.1)	2298 (87.1)	2056 (87.5)	1931 (88)	0.211
Foreigner	401 (13.9)	229 (12.9)	294 (12.5)	264 (12)	
*Marital status*					
Single	2298 (79.7)	2203 (83.6)	1896 (80.7)	1811 (82.6)	0.014
Married	546 (19)	405 (15.4)	426 (18.1)	361 (16.4)	
Widowed	2 (0.1)	0 (0)	2 (0.1)	1 (0)	
Separated	11 (0.4)	6 (0.2)	14 (0.6)	11 (0.5)	
Divorced	22 (0.8)	21 (0,8)	11 (0.5)	11 (0.5)	
*Level of education*					<0.001
Without studies	89 (3.1)	Not Registered	58 (2.5)	34 (1.5)	
Primary	348 (12.1)		200 (8.5)	131 (6)	
Secondary	683 (23.7)		618 (26.3)	603 (27.5)	
High School/VTE	1220 (43.4)		1015 (43.2)	992 (45.2)	
University	539 (18.7)		459 (19.5)	435 (19.8)	
*Social Class*					
Classes I and II	Not Registered	434 (17.1)	381 (17)	367 (17.5)	0.086
Classes III and IV		836 (33)	677 (30.1)	621 (29.5)	
Classes V and VI		1266 (49.9)	1187 (52.9)	1116 (53)	
*Body Mass Index*					
Insufficient weight	152 (5.5)	133 (5.3)	116 (5.1)	122 (5.7)	0.008
Normal weight	1722 (62.3)	1630 (64.8)	1501 (65.7)	1320 (61.8)	
Overweight	695 (25.2)	563 (22.4)	492 (21.6)	493 (23.1)	
Obesity	193 (7)	188 (7.5)	174 (7.6)	200 (9.4)	
*Self-perceived health status*					
Very good	973 (33.8)	967 (36.7)	895 (38)	873 (39.8)	0.004
Good	1595 (55.4)	1389 (52.6)	1174 (50)	1071 (48.8)	
Regulate/bad/very bad	311 (10.8)	281 (10.7)	281 (12)	251 (11.4)	

VTE, Vocational Education and Training.

**Table 2:** Frequency of physical activity in relation to health self-perception and sociodemographic and psychosocial characteristics in young Spanish adults aged 18–30 (2009–2017).

Characteristics	Sedentary n = 2464(34.4%)	Occasionally PA n = 1933(26.9%)	PA Several Times/Month n = 1458(20.3%)	PA Several Times/Week n = 1320(18.4%)	*p*
*Sex*					
Men	883 (35.8)	760 (39.3)	969 (66.5)	857 (64.9)	<0.001
Women	1581 (64.2)	1173 (60.7)	489 (33.5)	463 (35.1)	
*Nationality*					
Spanish	2066 (83.8)	1677 (86.8)	1325 (90.9)	1212 (91.8)	<0.001
Foreigner	398 (16.2)	256 (13.2)	133 (9.1)	108 (8.2)	
*Self-perceived health status*					
Very good	745 (30.2)	730 (37.8)	630 (43.2)	627 (47.5)	<0.001
Good	1347 (54.7)	979 (50.6)	713 (48.9)	594 (45)	
Regulate/bad/very bad	372 (15.1)	224 (11.6)	115 (7.9)	99 (7.5)	
*Depression*					
Yes	110 (4.5)	69 (3.6)	25 (1.7)	22 (1.7)	<0.001
No	2354 (95.5)	1863 (96.4)	1432 (98.3)	1298 (98.3)	
*Year Survey*					
2011/2012	1038 (42.1)	652 (33.7)	569 (39)	376 (28.5)	<0.001
2014	698 (28.3)	687 (35.5)	477 (32.7)	489 (36.6)	
2017	728 (29.5)	594 (30.7)	412 (28.3)	461 (34.9)	
*Consumption of alcohol*					
Daily	57 (2.3)	60 (3.1)	36 (2.5)	28 (2.1)	<0.001
3–6 times/week	88 (3.6)	86 (4.5)	80 (5.5)	53 (4)	
1–2 times/week	559 (22.7)	421 (21.8)	444 (30.5)	406 (30.8)	
1–3 times/month	948 (38.5)	831 (43)	634 (43.5)	598 (45.3)	
Teetotaler	812 (33)	534 (27.6)	263 (18.1)	235 (17.8)	
*Smoke*					
Yes	984 (39.9)	656 (34)	463 (31.8)	344 (26.1)	<0.001
No	1480 (60.1)	1274 (66)	994 (68.2)	976 (73.9)	
*Ability to concentrate*					
Better than usual	52 (3)	44 (3.5)	41 (4.2)	46 (5.5)	0.003
As usual	1503 (85.4)	1076 (86.6)	844 (86.1)	725 (86.8)	
Worse than usual	205 (11.6)	122 (9.8)	95 (9.7)	64 (7.7)	
*Loss of sleep due to worries*					
No, absolutely	721 (41)	502 (40.4)	428 (43.7)	445 (53.4)	<0.001
No more than normal	679 (38.6)	483 (38.9)	396 (40.4)	247 (29.6)	
More than normal	360 (20.5)	257 (20.7)	156 (15.9)	142 (17)	
*Ability to decide*					
More than usual	126 (7.2)	109 (8.8)	84 (8.6)	89 (10.7)	<0.001
As usual	1541 (87.6)	1095 (88.2)	859 (87.7)	727 (87.1)	
Less than usual	93 (5.3)	38 (3.1)	36 (3.7)	19 (2.3)	
*Ability to face problems*					
More than usual	96 (5.5)	77 (6.2)	46 (4.7)	67 (8)	<0.001
As usual	1560 (88.7)	1106 (89)	896 (91.5)	754 (90.4)	
Less than usual	103 (5.9)	59 (4.8)	37 (3.8)	13 (1.6)	
*Loss of self-confidence*					
No, absolutely	1225 (69.6)	908 (73.1)	707 (72.1)	658 (78.8)	<0.001
Equal o more than normal	536 (30.4)	334 (26.9)	273 (27.9)	177 (21.2)	
*Feeling happy*					
More than usual	153 (8.7)	123 (9.9)	79 (8.1)	112 (13.4)	<0.001
As usual	1494 (84.8)	1062 (85.5)	871 (89.1)	691 (82.8)	
Less than usual	114 (6.5)	57 (4.6)	28 (2.9)	32 (3.8)	

PA, physical activity.

**Table 3:** Body mass index related to sociodemographic and psychosocial variables, health status, alcohol consumption and frequency of physical activity in young Spanish adults aged 18–30 (2009–2017).

Characteristics	Insufficient Weight n = 523(5.4%)	Normal Weight n = 6173(63.7%)	Overweight n = 2243(23.1%)	Obesity n = 755(7.8%)	*p*
*Sex*					
Men	101 (19.3)	2831 (45.9)	1414 (63)	384 (50.9)	<0.001
Women	422 (80.7)	3342 (54.1)	829 (37)	371 (49.1)	
*Nationality*					
Spanish	469 (89.7)	5425 (87.9)	1910 (85.2)	658 (87.2)	0.003
Foreigner	54 (10.3)	748 (12.1)	333 (14.8)	97 (12.8)	
*Age*					
18–22	263 (50.3)	2174 (35.2)	527 (23.5)	165 (21.9)	<0.001
23–26	145 (27.7)	1864 (30.2)	629 (28)	201 (27.8)	
27–30	115 (22)	2135 (34.6)	1087 (48.5)	380 (50.3)	
*Marital status*					
Single	470 (89.9	5204 (84.3)	1704 (76)	539 (71.4)	<0.001
Married	49 (9.4)	894 (14.5)	513 (22.9)	212 (28.1)	
Wid/Sepa/Divor	4 (0.8)	73 (1.2)	26 (1.2)	4 (0.5)	
*Social Class*					
Class I and II	78 (22.4)	811 (19)	218 (14.5)	48 (9)	<0.001
Class III and IV	118 (33.9)	1360 (32)	435 (29)	165 (30.8)	
Class V and VI	152 (43.7)	2090 (49)	847 (56.5)	323 (51.3)	
*Self-perceived health status*					
Very good	178 (34)	2418 (39.3)	773 (34.5)	197 (26.1)	<0.001
Good	274 (52.4)	3135 (50.8)	1208 (53.9)	428 (56.7)	
Regulate/bad/very bad	71 (13.6)	610 (9.9)	262 (11.6)	130 (17.2)	
*Depression*					
Yes	17 (3.3)	155 (2.5)	73 (3.3)	44 (5.8)	<0.001
No	506 (96.7)	6017 (97.5)	2170 (96.7)	711 (94.2)	
*PA frequency*					
Sedentary	156 (42)	1377 (31)	538 (34.9)	269 (47.9)	<0.001
Occasionally PA	92 (24.8)	1211 (27.2)	387 (25)	170 (30.2)	
PA several times/month	70 (18.9)	952 (21.4)	339 (21.9)	66 (11.7)	
PA several times/week	53 (14.3)	907 (20.4)	282 (18.2)	57 (10.1)	
*Consumption of alcohol*					
Daily	11 (2.1)	139 (2.3)	75 (3.4)	13 (1.7)	<0.001
3–6 times/week	17 (3.3)	266 (4.3)	109 (4.9)	15 (2)	
1–2 times/week	91 (17.6)	1364 (22.3)	521 (23.5)	121 (16.2)	
1–3 times/month	262 (50.8)	2893 (47.2)	947 (42.7)	330 (44.2)	
Teetotaler	135 (26.2)	1468 (2I)	566 (25.5)	268 (35.9)	
*Anxiety*					
Yes	29 (5.5)	249 (4)	100 (4.5)	55 (7.3)	<0.001
No	494 (94.5)	5921 (96)	2142 (95.5)	700 (92.7)	
*Weight*					
<50 kg	332 (63.5)	430 (7)	1 (0)	0 (0)	<0.001
51–70 kg	191 (36.5)	4363 (70.7)	468 (20.9)	4 (0.5)	
71–90 kg	0 (0)	1367 (22.1)	1530 (68.2)	315 (41.7)	
91–110 kg	0 (0)	13 (0.2)	242 (10.8)	322 (42.6)	
>110 kg	0 (0)	0 (0)	2 (0.1)	114 (15.1)	
*Feel that you are worth nothing*					
No, absolutely	200 (78.7)	2418 (82.2)	834 (79.2)	300 (77.3)	0.001
No more than normal	46 (18.1)	457 (15.5)	185 (17.6)	64 (16.5)	
More than normal	8 (3.1)	68 (2.3)	34 (3.2)	24 (6.8)	
*Feeling unhappy*					
No, absolutely	132 (52)	1829 (62.1)	638 (60.6)	210 (54.1)	0.001
Equal o more than normal	122 (48)	1116 (37.9)	415 (39.4)	178 (45.9)	

Wid, widowed; Sepa, Separated; Divor, Divorced; PA, physical activity.

**Table 4:** Logistic regression model for the association between the frequency of physical activity, BMI and sociodemographic and psychosocial characteristics of young Spanish adults aged 18–30 (2009–2017).

	OR (95% CI)	*p*
*Body Mass Index*		
Insufficient weight	1.24 (0.71–2.17)	0.458
Normal weight	1.66 (1.25–2.20)	0.001
Overweight/Obesity	Reference	
*Consumption of alcohol*		
Daily	1.21 (0.62–2.35)	0.577
3–6 times/week	0.966 (0.59–1.58)	0.890
1–2 times/week	1.97 (1.47–2.63)	<0.001
1–3 times/month	1.56 (1.22–2)	<0.001
Teetotaler	Reference	
*Sex*		
Male	2.63 (2.09–3.32)	<0.001
Female	Reference	
*Marital status*		
Single	2.11 (1.58–2.8)	<0.001
Married/Widowed/Separated/Divorced	Reference	
*Level of education*		
Without education	Reference	
Primary	1.04 (0.36–3.00	0.944
Secondary	1.59 (0.60–4.23)	0.351
High School/VTE	1.98 (0.75–5.23)	0.169
University	2.66 (0.99–7.18)	0.054
*Feeling happy*		
More than usual	1.97 (1.09–3.58)	0.026
As usual	1.55 (0.91–2.66)	0.110
Less than usual	Reference	
*Social class*		
Classes I and II	1.34 (1.02–1.78)	0.042
Classes III and IV	1.35 (1.08–1.70)	0.008
Classes V and VI	Reference	
*Smoke*		
Yes	Reference	
No	1.66 (1.33–2.08)	<0.001
*Weight*		
<50 kg	1.11 (0.41–2.99)	0.844
51–70 kg	1.18 (0.48–2.91)	0.717
71–90 kg	1.98 (0.83–4.70)	0.122
91–110 kg	1.60 (0.65–3.99)	0.310
>110 kg	Reference	

VTE, Vocational Education and Training.
